# Providing Medical Information to Older Adults in a Web-Based Environment: Systematic Review

**DOI:** 10.2196/24092

**Published:** 2021-02-09

**Authors:** Bianca McLean, Nazia Hossain, Valentina Donison, Mikaela Gray, Sara Durbano, Kristen Haase, Shabbir Muhammad Husayn Alibhai, Martine Puts

**Affiliations:** 1 Michael G DeGroote School of Medicine McMaster University Hamilton, ON Canada; 2 Department of Internal Medicine, University of Toronto Toronto, ON Canada; 3 Lawrence S Bloomberg Faculty of Nursing University of Toronto Toronto, ON Canada; 4 Gerstein Science Information Centre University of Toronto Toronto, ON Canada; 5 University Health Network Toronto, ON Canada; 6 School of Nursing University of British Columbia Vancouver, BC Canada; 7 Department of Medicine, Institute for Health Policy, Management and Evaluation University of Toronto University Health Network Toronto, ON Canada

**Keywords:** eHealth, systematic review, geriatric assessment, geriatric oncology

## Abstract

**Background:**

Cancer is a disease that predominantly affects older adults, and several organizations recommend the completion of a geriatric assessment to help with cancer treatment decision-making. Owing to a shortage of geriatric teams and the vast number of older adults diagnosed with cancer each year, a web-based geriatric assessment may improve access to geriatric assessment for older adults. We systematically reviewed the literature to obtain the latest evidence for the design of our web-based geriatric assessment tool Comprehensive Health Assessment for My Plan.

**Objective:**

This review aimed to probe the following questions: what is the impact of providing health test results to older adults in a web-based environment without the presence of a health care provider for patient-centered outcomes, including satisfaction, perceived harm, empowerment, quality of life, and health care use (eg, hospitalization, physician visits, emergency room visits, and costs), and what recommendations do older adults and developers have for designing future apps or websites for older adults?

**Methods:**

This systematic review was guided by the PRISMA (Preferred Reporting Items for Systematic Reviews and Meta-analysis) statement. Studies were limited to publications in English that examined a web-based tool that provided test results to older adults (aged ≥65 years) without the presence of a health care provider. A health sciences librarian performed the search on November 29, 2019, on the following electronic databases: MEDLINE, Embase, CINAHL, PsycINFO, and the Cochrane Library. The quality of the included studies was assessed using the Mixed Methods Appraisal Tool Version 2018. The findings are summarized narratively and in tabular format.

**Results:**

A total of 26,898 titles and abstracts were screened by 2 independent reviewers, of which 94 studies were selected for a full-text review, and 9 studies were included in this review. There were only 2 randomized controlled trials of high quality that explored the effects of receiving health care results on the web via eHealth tools for older adults or provided evidence-based recommendations for designing such tools. Older adults were generally satisfied with receiving screening results via eHealth tools, and several studies suggested that receiving health screening results electronically improved participants’ quality of life. However, user interfaces that were not designed with older adults in mind and older adults’ lack of confidence in navigating eHealth tools proved challenging to eHealth uptake and use. All 9 studies included in this systematic review made recommendations on how to design eHealth tools that are intuitive and useful for older adults.

**Conclusions:**

eHealth tools should incorporate specific elements to ensure usability for older adults. However, more research is required to fully elucidate the impact of receiving screening and results via eHealth tools without the presence of a health care provider for patient-centered outcomes in this target population.

## Introduction

### Background

For older adults with cancer, several organizations recommend the completion of a geriatric assessment to help with cancer treatment decision-making [1,2]. A geriatric assessment consists of several questionnaires and tests that assess the medical, social, and psychological functioning of older adults to determine what interventions could be implemented to optimize their health and well-being [3]. However, owing to the shortage of geriatric teams and the large number of older adults diagnosed with cancer each year, access to a geriatric assessment remains to be limited. A web-based geriatric assessment may improve access for older adults. Although a few web-based geriatric assessment tools have been developed [4-6], these tools do not provide older adults with their test results without a health care provider being present. In addition, these tools would not increase access to a geriatric assessment because they still require the input of health care professionals, who are currently in low supply and high demand. Our overarching aim is to review the literature to develop a web-based geriatric assessment, the Comprehensive Health Assessment for My Plan (CHAMP), which will provide test results directly to older adults and help triage patients who are in greater need of geriatric consultation. To best design the CHAMP tool, we were interested in understanding the impact of receiving health test results in a web-based environment without the presence of a health care provider on older adults. We were also interested in consolidating the recommendations made by older adults and website developers for designing web-based tools for older adults. Therefore, we systematically reviewed the literature to obtain the latest evidence to inform the future design of our CHAMP tool.

As older adults with multiple comorbidities make up an increasing proportion of the population, there is a growing focus on equipping these patients with the tools needed to manage their own health. The aim is to provide patients with a sense of control over their medical conditions and decrease health care utilization [7]. Older patients particularly value the ability to manage their health independently at home, and minimizing reliance on health care resources, such as emergency rooms and inpatient units, is therefore an important outcome measure [8-10]. One strategy to meet these needs is the development of web-based health management tools that can be linked to patients’ eHealth records and accessed from personal devices (such as smartphones, tablets, and laptops). A wide variety of eHealth tools have been developed [5-7,11]). For example, some enable patients to view results of laboratory and imaging tests [11], whereas others provide customized health care advice or allow patients to communicate directly with members of their health care team [12]. Web-based tools have also been developed for the management of specific medical conditions such as cardiovascular disease [13] and diabetes [14]. The adoption of these resources was found to improve patient outcomes in these studies. In a small study of 169 computer users aged 50 years and older, Zettel-Wattson and Tsukerman [15] discovered that 90% of participants found patient portals helpful for managing their health and 80% felt that portals gave them control over their health. A systematic review by Ferreira et al [16] showed that providing patients access to their electronic medical records improved patient understanding of their disease and helped break down barriers in the physician-patient relationship. 

Despite the number of eHealth tools and their potential to enhance patient care, barriers exist to widespread adoption, especially among patients older than 65 years. Previous studies have cited concerns about privacy and security, lack of access to technology, low computer literacy, high computer anxiety, complex user interfaces, and concerns about losing face time with health care providers as key factors that prevent older adults from routinely using eHealth management systems [7,17-20]. Disparities in uptake have also been found based on age group, ethnicity, education level, and physical and cognitive abilities [7,18,21]. 

Studies have varied in their conclusions about optimal eHealth tool design, and few have offered specific recommendations to address these barriers. Some authors suggest that complete medical records, medication lists, test results, and condition-specific health advice are consistently appreciated by patients accessing web-based portals [15,17,22]. Khan et al [23] studied perceptions of a medication management system and found that participants enjoy visual representations of data but would also like accompanying text descriptions to fully understand their meaning. Furthermore, some patients desire the ability to receive appointment reminders, refill medications, or communicate with health care professionals through secure messaging. However, the impact of various designs on patient-centered outcomes remains to be fully explored [24].

### Objectives

To best design the CHAMP tool to deliver geriatric assessment results to older adults with cancer, our review questions were as follows:

1. What is the impact of websites and apps providing health test results to older adults in a web-based environment without the presence of a health care provider for patient-centered outcomes such as satisfaction, empowerment, quality of life, and health care use (eg, hospitalization, physician visits, emergency room visits, and costs)?
2. What recommendations do older adults and developers have for designing future apps or websites for older adults? 

We were most interested in understanding the impact of receiving health care screening and test results in the electronic environment on patient-centered outcomes such as satisfaction, empowerment, and quality of life compared with cancer-specific outcomes such as progression-free survival because we expect that the results of this literature review will be applicable to the care of older adults in many other fields of medicine, not just oncology. Furthermore, in geriatric oncology, factors other than progression-free survival and other cancer-specific outcomes are of substantial importance. Quality of life, overall functioning, and health care use have become increasingly important from the patient’s viewpoint. Hence, it is both of service to the patients that we care for, and to other providers of care for older adults to understand the impact of receiving health results in a web-based environment from the patient perspective [25,26].

### Methods 

#### Review Methodology

We used systematic review methodology according to the Cochrane Handbook [27] and guided by the Preferred Reporting Items for Systematic Reviews and Meta-analyses (PRISMA) statement [28]. 

#### Search Methods

Database searches were conducted by a health sciences librarian (MG) in Ovid MEDLINE, Ovid Embase, EBSCO CINAHL Plus with Full Text, Ovid PsycINFO, and the Cochrane Library using the Wiley interface. A combination of database-specific subject headings and text word searches was used to search for concepts included in our population intervention comparator outcomes search with publication date limits applied to identify articles published in the last 10 years. Keywords included “telehealth,” “eHealth” or “mHealth” or “mobile health” or “digital health” or “telecommunications” or “electronic mail” or “cell phone” or “smartphone” or “Internet” or “Mobile Applications,” “older adults,” and “aged.” Although a geriatric assessment is not the same as a patient portal (the former is a health assessment, whereas the latter is a web-based shared medical record), we expanded the search to include portals to identify any studies that looked at the impact of providing test results web-based on older adults’ health outcomes. The results of this search were imported with other search results. Published filters were applied to limit the publication type to randomized controlled trials (RCTs) [29-32]. See Multimedia Appendix 1 for the MEDLINE search. The searches were run on November 29, 2019, and the search period was from January 1, 2009, to November 29, 2019. The search period was limited to 2009 onward to ensure that any apps and website or design recommendations would still be relevant as eHealth is a rapidly developing field. Publications in English were eligible for inclusion. Reference lists of included studies were reviewed to identify any additional relevant studies. 

Papers were included if the following criteria were met:

- Included a population of older adults (aged above 65 years or the mean or median age in the study population was above 65 years, or if younger, subgroup analysis of those above 65 years was reported)
- Included an intervention in which older adults received results of health screening or tests completed in a web-based environment or eHealth (not including live chats with nurses, therapists, or doctors to go over test results)
- Compared the intervention to receiving the results of tests or screening in person from a health care provider or had no control group
- Focused on the following intervention outcomes: (1) patient-centered outcomes such as satisfaction, perceived harm, anxiety, depression, distress, empowerment, and quality of life; (2) health care use (eg, hospitalization, physician visits, emergency room visits, and costs); and (3) patient understanding of instructions of the tool used or provided recommendations on how to design eHealth tools for older adults

#### Study Selection

**Figure 1:**
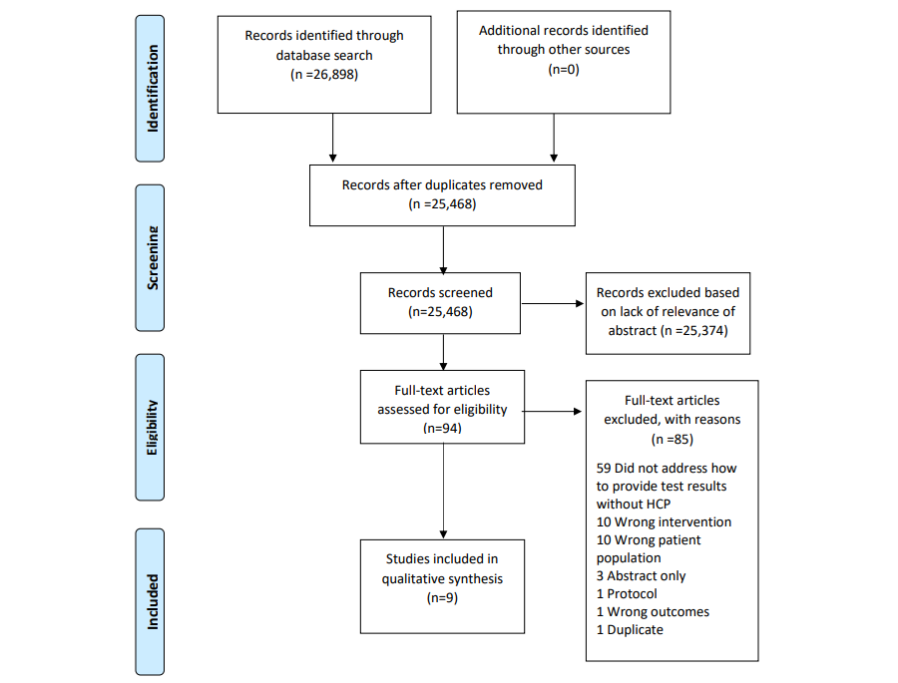
PRISMA (Preferred Reporting Items for Systematic Reviews and Meta-analyses) flowchart for study selection.

We included studies through a two-step process (see Figure 1 for our PRISMA flowchart). First, abstracts and titles were screened by two independent reviewers. Then, all potentially relevant full-text articles were reviewed for study inclusion by two independent reviewers. We used the Covidence software [33] to facilitate the study selection process. In case of disagreements, a third reviewer reviewed the abstract or full text, and a consensus decision was made whether to include or exclude the study.

#### Data Abstraction

We used standardized data collection forms developed by the research team using Excel. Data were abstracted by two reviewers independently and compared. The information that was abstracted included characteristics of the study population, study design details, details of the intervention (app or website), the methodology used to develop the app or website, details on the app or website, the impact of receiving web-based results for patients (on the aforementioned patient-centered outcomes), and details on the analyses used. For papers referring to a published study protocol, we obtained the study protocol paper to obtain the full methodological details of the study. After data abstraction, we had the missing information from all 9 studies. We contacted the authors of all the studies via email to inquire about missing information, and authors of 4 studies responded. As the studies were heterogeneous in design, intervention delivered, and outcome measures used, we summarized the abstracted data qualitatively because a meta-analysis was not possible.

#### Quality Assessment

We assessed the quality of the included studies using the Mixed Methods Appraisal Tool (MMAT) version 2018 [34-36]. MMAT is a quality assessment instrument that is useful for assessing qualitative, quantitative, and mixed methods studies. We noticed that several studies included a qualitative component (eg, multimethods and mixed methods studies); therefore, we chose to use MMAT over Cochrane Risk of Bias tool, which is not able to review these qualitative components. We used MMAT to review study quality, but we did not exclude any study based on the score as our aim was to understand all the evidence that was available and use that for our development of a web-based geriatric assessment.

#### Data Analysis

We summarized the results using a narrative descriptive synthesizing approach. A pooled analysis was not conducted because of heterogeneity in study inclusion criteria, interventions, and outcomes.

## Results

### Description of Included Studies

Of the 9 studies included in this review, 4 were qualitative studies [37-40], 2 were RCTs [41,42], 2 were mixed methods studies [43,44], and 1 was a quasi-experimental controlled study [45]. Overall, 8 studies were conducted in the United States [37-42,44,45], whereas 1 was a multinational study conducted in Western Europe [43]. All 9 studies included in this systematic review were published between 2015 and 2019. The sample size of the studies ranged considerably, with qualitative studies ranging from 24 to 44 participants [37-40] and the RCTs ranging from 50 to 272 participants [41-43]. The mixed methods study ranged from 88 (47 for the focus group and 41 for the pilot trial) [43] to 123 participants (23 for the focus group and 100 for the phone survey) [44], whereas the quasi-experimental study had 200 participants [45]. In addition, 4 studies evaluated the attitudes and experiences of older adults with patient portals [38,39,44,45], 2 studies tested web-based apps developed to deliver condition-specific (eg, cancer, cardiovascular disease) interventions [37,43], 1 study tested a user interface for a home health website [40], 1 tested a web-based decision aid [42], and 1 tested a theory-based patient portal training program [41]. A summary of the characteristics of each study included in this systematic review is shown in Table 1.

**Table 1:** Description of the included studies.

Study (reference)	Study design	Location	Sample size; population	Average age (years)	Female (%)	Sampling	Intervention app or tool	Analysis
Alpert et al (2016) [39]	Qualitative	United States	31 patient interviews; 2 focus groups of 13 health care professionals	Not reported (range 18-79)	58	Convenience	My preventative care patient portal	Critical incident technique
Baier et al (2015) [40]	Qualitative	United States	13 home health consumers; 28 case managers	71% ≥65 years; mean not reported	85	Convenience	Home health web-based app user interface	Content analysis
Irizarry et al (2017) [44]	Mixed methods	United States	100 older adults in phone survey, 23 in focus group	Focus group: 73; phone survey: 77	Focus group: 52.2%; phone survey: 46.2%	Convenience	Patient portal	Thematic analysis, Kruskal Wallis rank test, chi-square test
Jongstra et al (2017) [43]	Pilot RCT^a^	Western Europe	41 older adults with elevated CVD^b^ risk	69	56	Random	Web-based app (HATICE) for older adults with CVD risk	Descriptive statistical analysis
Loh et al (2018) [37]	Qualitative	United States	18 older adults with malignancy; 13 caregivers	Patient: 77; caregiver: 70	Patient: 17; caregiver: 92	Convenience	TouchStream app to deliver geriatric oncology interventions	Conventional content analysis
Nahm et al (2019) [41]	RCT	United States	272 older adults with chronic disease	70	70.2	Convenience	Theory-based patient portal e-learning program	Linear mixed model, *t* test, chi-square test
Portz et al (2019) [38]	Qualitative	United States	24 older adults with chronic disease	78	71	Stratified	Kaiser permanente colorado’s patient portal—my health manager	Theoretical analysis based on the technology acceptance model
Smallwood et al (2017) [42]	Pilot RCT	United States	50 older women with BMD^c^ indicating osteopenia or osteoporosis	Median 79 years; mean not reported	100	Stratified	Decision aid within patient portal for osteoporosis	ANOVA, *t* test, chi-square test
Toscos et al (2016) [45]	Quasi-experimental controlled	United States	200 patients with significant CAD^d^	Average age not reported; 58% older than 66 years	27.5	Not reported	Personal health record	Linear regression, *t* test, Cochran-Mantel-Haenszel test

^a^RCT: randomized controlled trial.

^b^CVD: cardiovascular disease.

^c^BMD: bone mineral density.

^d^CAD: coronary artery disease.

### Quality of Studies Included

The application of MMAT to each study included in this review is shown in Multimedia Appendix 2. Overall, there were no studies of high quality that looked at the effect of web-based screening without the presence of a health care provider on older adults or evidence-based eHealth design. Most studies that were included had small sample sizes [42-44] and used convenience sampling [37,39-41,44], thereby increasing the risk of selection bias. We were unable to determine if the outcome assessors were blinded in all RCTs [41,42], and we were unsure how randomization was performed in one of the RCTs [42]. In half of the qualitative studies included in this systematic review, we were unable to determine if there was coherence between the qualitative data source, analysis, and interpretation [37,40].

The results of our systematic review are stated in the order of our aims. First, we review our findings on how receiving health screening in a web-based environment affects satisfaction, perceived harm, quality of life, and health care utilization by older adults. Second, we consolidate evidence-based recommendations on how to design eHealth tools that are useful and engaging for older adults.

### Objective 1: Effects of Health Screening in a Web-Based Environment

A total of 7 studies in this review evaluated the effects of receiving health screening tests or results in a web-based environment without the presence of a health care provider on older adult participants’ satisfaction (n=6), perceived harm (n=5), and quality of life (n=5) [37-39,41,42,44,45]. No studies included in this review reported on the effect of eHealth tools on health care use by older adults. The main findings of these studies are shown in Table 2. Screening results from eHealth tools were generally well received by older adults, but several studies suggested that older adults felt anxious about using new technology [37,38,41,44]. In total, 63% of patients in the study by Loh et al [37] found the TouchStream health app, used to deliver geriatric interventions to older adults with cancer, enjoyable to use. A total of 20 participants (87%) in the study by Irizarry et al [44] felt that patient portals were generally useful. Five physicians (56%) in the study by Alpert et al [39] suggested that the investigated patient portal improved patient empowerment. Participants in the study by Portz et al [38] indicated that the Kaiser Permanente patient portal improved patient-provider communication and saved patients time and money. Older women with osteoporosis felt more prepared to make treatment decisions after using the web-based decision-making tool designed and studied by Smallwood et al [42]. Most participants in the same study [42] were able to complete the web-based decision aid, although 5 participants (17%) entered the information incorrectly.

**Table 2:** Effects of receiving health information in web-based environment for older adults.

Study	Satisfaction	Perceived harm	Quality of life	Health care use
Alpert et al (2016) [39]	Patients found the portal useful for instantly accessing medical information. This feature accounted for more than half of the positive incidents recorded. Patients appreciated receiving laboratory test	A total of 11% of negative incidents were because of patients having difficulty interpreting laboratory test results. Patients were concerned when information was incorrect or not updated. There were more negative incidents (n=82, 72.6%) than positive incidents (n=31, 27.4%)	Physicians (n=5, 56%) suggested that the portal made patients feel empowered	NS^a^
Irizarry et al (2017) [44]	A total of 87% (n=20) of participants generally felt that the patient portal was useful. Participants with both low and high health literacy expressed interest in portal training. Participants who had experienced chronic illness praised the convenience of web-based laboratory results	57% of participants (n=13) had anxiety and frustrations about using technology because of their perceived lack of technological skills. This caused them to rely on family members to use the patient portal	NS	NS
Loh et al (2018) [37]	Most patients (n=10, 63%) and caregivers (n=8, 73%) enjoyed using the eHealth app to connect with their care providers and manage their health. Most patients or caregivers found the health app easy to use	One patient (6%) suggested that the app may be difficult for someone with less experience using technology	25% (4/16) of patients commented that the app would be most useful for patients living alone	NS
Nahm et al (2019) [41]	NS	NS	Patient portal training improved user health decision-making, patient-provider communication, and eHealth literacy. At 4 months after patient portal training, changes in self-efficacy (*P*=.02) and patient portal usage (*P*=.03) were significant	NS
Portz et al (2019) [38]	Users suggested the patient portal was useful for accessing health information and communicating with their health care providers	Users were anxious that program updates would cause the portal to become unfamiliar or too difficult to use	Users believed the patient portal saved them time and money	NS
Smallwood et al (2017) [42]	Participants were able to complete the web-based decision aid with minimal assistance. Subjects who used the decision aid compared with those who did not use it felt more prepared to make decisions about their treatment (*P*<.001)	Some patients (n=5, 17.2%) incorrectly entered information into the decision tool	NS	NS
Toscos et al (2016) [45]	The mean activation of participants was of the highest possible level (level 4) throughout the study	NS	Patient activation was higher in portal users, but not statistically significant. Portal users showed health improvements at 12 months in HbA_1c_^b^, LDL^c^, SBP^d^, and DBP^e^, but only HbA_1c_ (−0.19; *P*=.005) was statistically significant. BMI was unchanged throughout the study	NS

^a^NS: not studied.

^b^HbA_1c_: glycated hemoglobin A1c.

^c^LDL: low-density lipoprotein.

^d^SBP: spontaneous bacterial peritonitis.

^e^DBP: diastolic blood pressure.

Although participants were generally positive about the use of eHealth to receive screening or test results, several studies noted that older adults reported feeling anxious about using eHealth technology [38,39,44]. Participants—especially those with low health literacy—felt afraid to make mistakes because of their lack of technological experience. Many of these patients commented that computer use was not common in their working environment, which accounted for their lack of experience. These participants often avoided technology use altogether and preferred a family member accessing their patient portal on their behalf [44]. Participants in the study by Portz et al [38] noted specific anxiety about program updates to eHealth tools that made eHealth tools difficult to use after patients had learned and were comfortable with the tools. Difficulty in interpreting and applying laboratory results was also a concern among older eHealth users [39]. However, several studies noted that patients still enjoyed being able to view their laboratory results on the web [38,39].

Despite computer anxiety being common among this population, many older adults, including those with low health literacy, were still interested in learning how to use a patient portal [44]. Patient portal training may be an important solution to low confidence that prevents many older adults from utilizing patient portals. Nahm et al [41] conducted an RCT and found that a theory-based patient portal e-learning program resulted in statistically significant improvements in patient portal self-efficacy, health decision-making, patient-provider communication, and eHealth literacy 3 weeks after portal training. Patient portal self-efficacy remained significantly higher in the intervention group at 4 months [41]. Participants from several studies recommended providing an instructional video or detailed written instructions to aid platform navigation [40,43]. Participants with both high and low health literacy felt that task-based training programs were a valuable but underutilized tool to increase confidence and knowledge on how to navigate eHealth tools [44].

### Objective 2: Designing eHealth Tools for Older Adults

All 9 studies included in this review provided recommendations on how to develop eHealth tools that are intuitive, useful, and engaging for older adults. The specific recommendations can be divided into 3 basic categories: (1) user interface (how the participant interacts with the eHealth tool), (2) functionality (what the participant wants the eHealth tool to do), and (3) information included (what the participant wants the eHealth tool to say). A summary of the recommendations can be found in Table 3.

**Table 3:** Older adult and investigator recommendations for eHealth tools.

Theme and study			Older adult recommendation		Investigator recommendation
**User interface**					
	Alpert et al (2016) [39]	Write information as bulleted lists Dictionary to look up challenging terminology		Create an interactive user interface Use images that represent the information being presented Use motivational voice, not passive voice	
	Baier et al (2015) [40]	If the page requires scrolling to view all the content, add a pop-up to remind the user to scroll down Allow users the option to increase font size Results and health information should be easily printed The web-based apps should be optimized for mobile devices		Avoid writing in all caps Use serif fonts Use contrasting colors to enhance readability Provide prompts for functions Write at a sixth-grade reading level, limit technical language Include definitions for medical terms Directly label graphs Limit comparisons with 3-4 points	
	Jongstra et al (2017) [43]	Use language that focuses on health rather than disease Log-in passwords should not be complicated Include interactive features Health information should be easily printed		Use large font size Use simple and consistent layout with large buttons Use images and distinct colors to facilitate page navigation Include audio option	
	Loh et al (2018) [37]	N/A^a^		Ensure reliable internet access Provide stylus for touchscreen devices Provide a list of voice options if audio included Optimize the app for mobile phones and tablets Ensure screen brightness, font and color are easily readable	
	Portz et al (2019) [38]	Use larger font and contrasting colors		N/A	
	Smallwood et al (2017) [42]	N/A		Automatic entry of patient’s lab scores to decrease incorrect information	
**Functionality**					
	Alpert et al (2016) [39]	Ability to communicate with the physician regarding information received on the portal Seamless and intuitive password retrieval		Ability for physician to confirm if their patient viewed or understood the information provided to them	
	Baier et al (2015) [40]	Add detailed instructions at the beginning of the eHealth tool to help users learn how to navigate the tool		N/A	
	Irizarry et al (2017) [44]	Include task-based training to help users understand how to navigate the different features of the patient portal		Integrate the patient portal with in-person clinical encounters Allow personnel to edit missing or inaccurate information in the patient portal	
	Jongstra et al (2017) [43]	Provide a way for patients to ask questions about navigating the online platform Include an instructional video to aid in platform navigation		Include games, goal setting, automated messages among other interactive features to motivate eHealth use	
	Loh et al (2018) [37]	Participants found functions including appointments, medications, nutrition, and exercise reminders helpful		If symptom reporting is included, ensure that feedback is provided on reported symptoms Provide digital activity tracker when exercise intervention is recommended Incorporate nonmedical functions such as social activities, jokes, games, etc	
	Nahm et al (2018) [41]	N/A		Implement patient portal training for older adults	
	Portz et al (2019) [38]	Participants were interested in using e-visits and chat functions with providers		Portal designers should consider including functions that integrate eHealth with physical clinic visits	
**Information included**					
	Alpert et al (2016) [39]	Include personalized, not generic health information Patients appreciated receiving laboratory results but sometimes had difficulty interpreting them		N/A	
	Jongstra et al (2017) [43]	Provide practical and reliable health information		N/A	
	Loh et al (2018) [37]	N/A		Tailor interventions and activities to the individual	
	Toscos et al (2016) [45]	N/A		Apply a user-centered design approach to tailor the portal to the specific population that it is designed for	

^a^N/A: not applicable.

#### User Interface

A total of 7 studies made recommendations regarding how to design a user interface that is accommodating for older adults [37-40,42,43]. Most of the design recommendations suggested how to develop platforms that are easier to read and navigate. Recommendations included using a simple layout with large font, contrasting colors, and images that relate to the content [37-40]. Participants also wanted the technology to work seamlessly, with uncomplicated log-in, the ability to print information, and the ability to use the tool on smartphones and tablets [37,40,43]. Participants not only focused on the visuals and layout but also the tone, with recommendations for eHealth tools to use language that is motivating and positive and focuses on health rather than disease [39,43]. Finally, several studies recommended using an interface that is interactive to engage the user and encourage them to continue using the eHealth tool [39,43].

#### Functionality

A total of 7 studies identified functions that participants found most useful to be included in an eHealth tool [37-41,43,44]. A common suggestion among older adults was to include detailed instructions within the eHealth tool [20,40,43,44]. Several suggestions were given as to how instructions should be included. Participants in the study by Baier et al [40] recommended detailed written instructions accessible within the eHealth tool. Participants in the study by Irizarry et al [44] suggested that task-based training was most helpful for learning how to navigate the tool. Alternatively, instructional videos and communication methods that allowed participants to ask questions about navigating the platform were recommended by participants in the study by Jongstra et al [43].

Both authors and participants of 3 studies commented that the eHealth tool should be integrated with the in-person clinical environment [38,39,44]. Participants commonly cited the ability to communicate with their physician through the eHealth tool as an enjoyable and useful feature [38,39]. Physicians generally felt that the patient portal empowered patients, but they wanted the ability to confirm if their patient viewed and understood the information provided to them via the eHealth tool [39]. Portz et al [38] suggested using face to face or phone time to encourage portal use in patients.

Finally, 2 studies recommended including fun, interactive features such as games, jokes, social activities, or automated motivational messages to promote tool use and make the tool more enjoyable for older adults [37,43].

#### Information Included

A total of 4 studies made recommendations regarding which information older adults found most useful to include in an eHealth tool [37,39,43,45]. Two studies found that personalized health information is more useful and engaging for older adults than generic health information [37,39]. Toscos et al [45] suggested that applying a user-centered design approach to the development of eHealth tools may promote the inclusion of information that is more tailored to older adults. Participants also wanted practical and reliable health information included in the eHealth tool [43].

## Discussion

### Principal Findings

The aims of this systematic review were two-fold. First, we were interested in understanding how receiving health screening in a web-based environment without the presence of a health care provider affects satisfaction, perceived harm, quality of life, and health care use by older adults. Second, we were interested in consolidating evidence-based recommendations on how to design eHealth tools that are useful and engaging for older adults. We found that older adults generally had positive experiences with receiving test results via eHealth tools, and numerous features have been suggested to enhance patients’ web-based experiences. Although much literature is available on the impact of eHealth tools for younger patients, older adults represent a unique subgroup of patients whose needs differ greatly [46-48]. To the best of our knowledge, there are currently no systematic reviews on the effects of receiving health screening or results via eHealth tools either on older adults’ health care satisfaction, perceived harms, quality of life, or health care use or on the optimal design for eHealth tools for older adults. It is important to understand the unique experiences of older adults because they are often less proficient with technology than younger patients are and may require different supports [49]. As a rapidly growing population of health care consumers, older adults are positioned to benefit greatly from the use of eHealth tools if these tools are designed in ways that are attractive to older adults.

From the 9 studies included in our review, several key themes emerged. Multiple studies noted that while older adults were generally optimistic about eHealth tools, lack of technology experience and fear of failure were barriers to use [37,38,44]. Both older adults and researchers recommended detailed instructions and comprehensive training to improve older adults’ confidence in using eHealth tools [40,41,43,44]. Although it is encouraging that most older adults found receiving screening tests and results via eHealth to be useful, there is currently not enough research available to draw conclusions on the impact of receiving test results in a web-based environment without the presence of health care providers on older adult satisfaction, perceived harm, and quality of life. The possible harms of providing older adults with screening results via eHealth tools are anxiety caused by technology use, confusion among older adults who may be unable to interpret their results, and disparity caused by those who are less likely to benefit from eHealth tools because of low technology or health literacy. We were unable to find any information on the effect of eHealth screening tools on older adult health care utilization and hence cannot recognize any trends or draw any conclusion on health care use.

There were substantial recommendations from the studies included in this systematic review on how to design eHealth tools for older adults. A user interface that is accessible and intuitive to older adults is imperative for promoting tool uptake and use and was the most commonly made recommendation provided by older adults. Further recommendations included ensuring that the layout and text used in the tool is accessible to users with vision or hearing impairments and is logical to those with less technological experience. Furthermore, eHealth tools should be enjoyable for older adults to use. Designing a tool that includes interactive features, uses a positive tone, and ensures a seamless technological experience creates an environment that promotes eHealth tool uptake.

To promote eHealth use among older adults, the tools must provide functions and content that are useful for older adults. Participants emphasized the importance of integrating the eHealth tools with the physical clinic environment by facilitating communication with their physicians. Older adults suggested that personalized information, interventions, and activities were more useful and engaging than generic recommendations.

### How Does This Compare With the Literature?

Although there are several systematic reviews that investigate the effect of eHealth tools on healthy aging outcomes such as physical activity, diet, and psychological well-being [50-52], we were unable to find a systematic review that investigated the effects of receiving screening results without the presence of health care providers in older adults. Furthermore, we were unable to find a systematic review that consolidated evidence-based recommendations for designing eHealth tools for older adults. Kampmeijer et al [53] completed a systematic review on the use of eHealth tools in health promotion and primary prevention for older adults. Similar to our findings, Kampmeijer et al [53] found that usability and accessibility were important facilitating factors in older adults’ use of eHealth tools [53]. Buyl et al [52] completed a systematic review on the effect of eHealth interventions on healthy aging outcomes such as physical activity, psychological well-being, and overall health. Similar to our study, Buyl et al [52] were unable to draw conclusions on most health-related outcomes as they also found the quality of studies to vary considerably and the certainty of evidence to be low. However, Buyl et al [52] found that eHealth tool use improved older adults’ physical activity. Strengthening digital competency was a critical component of encouraging eHealth tool use among older adults, which is similar to our finding that older adults desire training programs to feel confident in using eHealth tools. However, our study differs from those by Buyl et al [52] and Kampmeijer et al [53] because both studies investigated eHealth tools that encouraged physical activity, psychological well-being, and primary prevention strategies for older adults, whereas we investigated tools that provided screening results to older adults without health care providers present. Furthermore, Narasimha et al [54] completed a systematic review of the optimal design of telemedicine for older adults. Encouragingly, the authors found that older adults were generally positive about their experience with telehealth, although a lack of confidence with technology and physical impairments (for example, hearing difficulty) proved to be a challenge. These results are similar to our findings that although older adults are optimistic and willing to use eHealth tools, designing tools that accommodate common physical impairments and include training are important for user confidence and uptake. Our systematic review is different from Narasimha et al [54] because we investigated evidence-based recommendations for developing eHealth tools, not telemedicine.

### Limitations

After removing duplicate and irrelevant papers, a small number of studies were used for our final analysis, which limits the generalizability of our findings. Although many titles and abstracts were found, we applied the RCT filter as we were interested in studies that examined the intervention ideally to a comparator group. However, few studies used an RCT design, and 4 studies used a qualitative design. By applying the RCT filter, it is possible that we may have missed additional qualitative studies. However, the gold standard for evaluating interventions is the RCT design, and these studies, including quasi-experimental studies, would have been identified in our search. Furthermore, most of the studies used convenience sampling to recruit participants, which introduces significant selection bias. In addition, the studies often had small sample sizes of less than 100 patients. These limitations further constrain the applicability of the results to larger and more diverse populations. Finally, many studies did not look at the sustainability of portal use, or the duration of follow-up was not reported. Therefore, it is unclear if any benefits that were identified are sustained over a significant period.

### Implications

Our findings suggest that although older adults are generally satisfied with receiving screening tests and results via eHealth tools, improper design, and lack of confidence with technology are common barriers to use in this population. Patients and caregivers should initially receive basic training on how to use eHealth tools to mitigate patient concerns (eg, about complex user interfaces) and minimize the impact of low computer literacy. To optimize the usability of eHealth tools, they should include customizable features (such as alerts, medication reminders, and appointment scheduling) as well as easy-to-read displays (eg, with large fonts and contrasting colors). In addition, eHealth tools should be integrated with physical clinic visits to facilitate communication between patients and their health care providers. By incorporating these features routinely into the design of patient portals, older adults will be more likely to embrace technology that can potentially improve their health. However, our review demonstrates that the literature on this topic remains sparse, and there is a need to further study the effects of eHealth tools on important patient-centered outcomes such as satisfaction, perceived harm, quality of life, and health care use. Older patients highly value the ability to remain at home, and avoiding emergency room visits and hospitalizations, making this an important outcome to consider in research involving older adults [8-10].

The findings from this systematic review will aid in the design of our CHAMP tool for older adults with cancer. Notably, one aim of this systematic review was to understand the impact of receiving test results in a web-based environment without the presence of health care providers on older adults. Although older adults generally appreciated receiving their results on the web, several studies noted that older adults desired the option to review their results with a health care professional. This supports our proposed CHAMP tool in which patients will use the tool to receive health care recommendations specific to their needs. Patients who are in high need of geriatric interventions will be identified and triaged to see a geriatrician. Those who are determined to have a low need for geriatric support will receive evidence-based recommendations determined by their unique health care needs and goals. These patients may also use the findings and recommendations of the CHAMP tool in discussions with their primary practitioner or oncologist. Hence, both low- and high-risk patients have the option to review and discuss the findings from the CHAMP eHealth tool with a health care professional.

The abundance of design recommendations made by older adults in the studies included in this systematic review will aid us in designing the CHAMP tool in a way that is most intuitive for older adults. Several design recommendations such as goal setting, live chat functions, and interactive games are more suited toward eHealth tools that are meant to be used longitudinally, whereas the CHAMP tool will be a one-time eHealth screening tool. However, these recommendations are still useful for researchers designing longitudinal eHealth tools for older adults.

### Recommendations for Future Research

The development and use of eHealth tools among older adults are an understudied area with an opportunity for more learning, particularly given the growing uptake of eHealth tools by older adults [55]. Currently, there is not enough research available to draw conclusions about the impact of receiving test results on the web on outcomes such as satisfaction, perceived harms, quality of life, and health care use for older adults. Future studies should investigate these outcomes in controlled trials that examine the impact of receiving test results on the web without a health care provider present. Future studies should also use random sampling methods that allow for greater generalization of the results. Finally, we were unable to find any research on the long-term implications of eHealth tools on the health and well-being of older adults or on health care use. Future studies should incorporate long-term follow-up and include health care use as an outcome to understand the extent of the benefits of eHealth tools.

## Appendix

Multimedia Appendix 1 MEDLINE search.

Multimedia Appendix 2 Quality assessment of included studies assessed with the Mixed Methods Appraisal Tool.

## Abbreviations

CHAMP: Comprehensive Health Assessment for My Plan
mHealth: mobile health
MMAT: Mixed Methods Appraisal Tool
PRISMA: Preferred Reporting Items for Systematic Reviews and Meta-analyses
RCT: randomized controlled trial
