# Supplementary material for: Providing Medical Information to Older Adults in a Web-Based Environment: Systematic Review
Source: JMIR Aging. 2021 Feb 9;4(1):e24092. doi: 10.2196/24092 (PMC8294635; doi:10.2196/24092)
Supplement: Multimedia Appendix 1 [file aging_v4i1e24092_app1.docx]

**Appendix 1 Medline search**

**Ovid MEDLINE: Epub Ahead of Print, In-Process & Other Non-Indexed Citations, Ovid MEDLINE® Daily and Ovid MEDLINE® <1946-Present>**

**Nov 29 2019**

| **#** | **Searches** | **Results** |
| --- | --- | --- |
| 1 | exp Aged/ | 3020981 |
| 2 | Geriatrics/ | 29570 |
| 3 | Aging/ | 224248 |
| 4 | Health Services for the Aged/ | 17391 |
| 5 | (ageing or aging or aged or elder* or geriatric* or old age* or senior* or older).tw,kf. | 1239892 |
| 6 | 1 or 2 or 3 or 4 or 5 | 3829352 |
| 7 | randomized controlled trial.pt. | 495253 |
| 8 | controlled clinical trial.pt. | 93439 |
| 9 | randomized.ab. | 461382 |
| 10 | placebo.ab. | 202887 |
| 11 | drug therapy.fs. | 2159915 |
| 12 | randomly.ab. | 322085 |
| 13 | trial.ab. | 484526 |
| 14 | groups.ab. | 1978905 |
| 15 | 7 or 8 or 9 or 10 or 11 or 12 or 13 or 14 | 4575292 |
| 16 | (animals not (humans and animals)).sh. | 4613662 |
| 17 | 15 not 16 | 3962818 |
| 18 | 6 and 17 [older adults and rcts] | 1087960 |
| 19 | Telemedicine/ | 20756 |
| 20 | (telemedicine or tele-medicine or telehealth or tele-health).tw,kf. | 14820 |
| 21 | (mhealth or m-health or mobile health or ehealth or e-health or electronic health or emedicine or e-medicine or digital health).tw,kf. | 27399 |
| 22 | Telenursing/ | 208 |
| 23 | (telenursing or tele-nursing).tw,kf. | 201 |
| 24 | telecommunications/ or electronic mail/ or cell phone/ or smartphone/ or Internet/ or Mobile Applications/ | 89541 |
| 25 | ((mobile adj3 phone*) or app or apps or application* or web or internet or tablet* or computer* or laptop* or iphone* or ipad* or smartphone* or smart phone* or cell phone* or cellular phone* or cellular device* or email* or e-mail* or website* or online or electronic mail).tw,kf. | 1694424 |
| 26 | exp Health/ | 349939 |
| 27 | exp Medicine/ | 1094199 |
| 28 | (health* or medicine or medical).tw,kf. | 3790528 |
| 29 | 26 or 27 or 28 | 4591339 |
| 30 | 24 or 25 | 1721047 |
| 31 | 29 and 30 | 380070 |
| 32 | 19 or 20 or 21 or 22 or 23 or 31 | 410154 |
| 33 | 18 and 32 | 23154 |
| 34 | limit 33 to (english language and yr="2009 -Current") | 16176 |
