# Supplementary material for: Providing Medical Information to Older Adults in a Web-Based Environment: Systematic Review
Source: JMIR Aging. 2021 Feb 9;4(1):e24092. doi: 10.2196/24092 (PMC8294635; doi:10.2196/24092)
Supplement: Multimedia Appendix 2 [file aging_v4i1e24092_app2.docx]

**Multimedia Appendix 2.** Quality assessment of included studies assessed with the Mixed Methods Appraisal Tool.

| Study (reference) | | Is the qualitative approach appropriate to answer the research question? | Are the qualitative data collection methods adequate to address the research question? | Are the findings adequately derived from the data? | Is the interpretation of the results sufficiently substantiated by data? | Is there coherence between qualitative data source, collection, analysis, and interpretation? |
| --- | --- | --- | --- | --- | --- | --- |
| **Qualitative study** | | | | | | |
|  | Alpert et al (2016) [39] | Yes | Yes | Yes | Yes | Yes |
|  | Baier et al (2015) [40] | Yes | Yes | Unsure | Unsure | Unsure |
|  | Loh et al (2018) [37] | Yes | Yes | Yes | Yes | Unsure |
|  | Portz et al (2019) [38] | Yes | Yes | Yes | Yes | Yes |
| Study | | Is the randomization appropriately performed? | Are the groups comparable at baseline? | Are there complete outcome data? | Are the outcome assessors blinded to the intervention provided? | Did the participants adhere to the assigned intervention? |
| **Randomized controlled trial** | | | | | | |
|  | Nahm et al (2019) [41] | Unsure | Yes | Yes | Unsure | Yes |
|  | Smallwood et al (2017) [42] | Yes | Yes | Yes | Unsure | Yes |
| Study | | Is there an adequate rationale for using mixed methods? | Are the different components of the study effectively integrated to answer the research question? | Are the outputs of the integrations of the qualitative and quantitative components adequately interpreted? | Are divergences and inconsistencies between quantitative and qualitative results adequately addressed? | Do the different components of the study adhere to the quality criteria of each tradition of the methods involved? |
| **Mixed methods** | | | | | | |
|  | Irizarry et al (2017) [44] | Yes | Yes | Yes | No | Yes |
|  | Jongstra et al (2017) [43] | Yes | Yes | Yes | Unsure | Yes |
| Study | | Are the participants representative of the target population? | Are the measurements appropriate regarding both the outcome and the intervention? | Are there complete outcome data? | Are the cofounders accounted for in the design and analysis? | During the study period is the intervention administered as intended? |
| **Quasi-experimental controlled** | | | | | | |
|  | Toscos et al (2016) [45] | Unsure | Yes | Yes | Yes | Yes |
